# OLDER ADULTS’ PERCEPTIONS AND ATTITUDES TOWARD TECHNOLOGIES TO ADDRESS LONELINESS: AN INTEGRATIVE REVIEW

**DOI:** 10.1093/geroni/igae098.0286

**Published:** 2024-12-31

**Authors:** Emma Cho, Charlotte Weiss, George Demiris

**Affiliations:** University of Pennsylvania, Philadelphia, Pennsylvania, United States; University of Pennsylvania, Philadelphia, Pennsylvania, United States; University of Pennsylvania, Philadelphia, Pennsylvania, United States

## Abstract

Older adults are at increased risk for loneliness and social isolation, which can affect healthy aging and well-being. This study aimed to understand how older adults perceive technology interventions that address loneliness and social isolation. Databases (PubMed, CINAHL, APApsychInfo, and Abstracts in Social Gerontology) were searched for articles reporting on perceptions of technologies that address social isolation and loneliness in older adults (65 years of age or older). Thirteen studies with English language, and peer-reviewed articles in various countries met the quality review and inclusion criteria. The findings of the review were synthesized into themes and sub-themes. The emergent themes suggest that older adults may positively perceive technologies (e.g., video phones, avatar pets, social assistive robots) to facilitate increased social connectedness, emotional attachment, companionship, and comfort, and connection to resources in the wider community. Older adults may also experience negative perceptions regarding technology: dissatisfaction with digital social communication style or content (e.g., repetitive conversations with digital pet) and technical barriers (e.g., audio delay on videophone, multiple steps to follow to use telephone). Technologies can offer solutions to address social connectedness in older adults, by mitigating loneliness and social isolation. Yet opportunities exist to partner with older adults to understand the barriers to using technology and co-create acceptable and feasible digital strategies to improve their overall well-being by reducing perceived loneliness and providing opportunities for improving social connectedness.

